# Agonistic autoantibodies targeting AT1R and ETAR: Mechanistic insights and emerging implications in cardiovascular disease

**DOI:** 10.1016/j.jtauto.2026.100371

**Published:** 2026-04-16

**Authors:** Francesco Tona, Giacomo Bernava, Giovanni Civieri

**Affiliations:** a Clinical Cardiology Unit, Department of Cardiac, Thoracic, Vascular Sciences and Public Health, University of Padua, Padua, Italy; b Cardiovascular Disease Modeling and Regenerative Medicine, Department of Cardiac, Thoracic, Vascular Sciences and Public Health, University of Padua, Padua, Italy

## Abstract

Autoimmune mechanisms are increasingly recognized as contributors to cardiovascular disease, extending beyond the traditional view of autoantibodies as passive markers of tissue injury. Among functional autoantibodies, those targeting the angiotensin II type 1 receptor (AT1R) and the endothelin-1 type A receptor (ETAR) represent a distinctive class capable of inducing sustained, ligand-independent activation of vasoactive G protein–coupled receptors.

Emerging experimental and translational evidence suggests that these agonistic autoantibodies may contribute to endothelial dysfunction, microvascular injury, and adverse cardiac remodelling through persistent receptor stimulation and downstream pro-inflammatory and pro-fibrotic signalling. Clinical observations, particularly in reperfused ST-elevation myocardial infarction, support an association with impaired myocardial reperfusion and unfavourable outcomes, although causality remains unproven.

In this perspective, we aim to delineate the mechanistic framework of AT1R- and ETAR-targeting agonistic autoantibodies, critically appraise the available clinical evidence, and outline key priorities for their translational validation in cardiovascular disease. Further investigation is required to determine their potential role in risk stratification and as therapeutic targets. Integrating immunological markers into cardiovascular research may provide new avenues for translational advancement, while necessitating rigorous validation in well-designed clinical studies.

## 1 Introduction

Autoantibodies are not new to cardiology. In dilated cardiomyopathy and myocarditis, immune responses against β1-adrenergic receptors, myosin, and other myocardial proteins have long been recognized [1]. In immune checkpoint inhibitor (ICI)–related myocarditis, immune-mediated mechanisms are clearly implicated, although the specific contribution of autoantibodies remains incompletely defined [2]. These findings indicate that autoimmune responses may precede and contribute to myocardial damage, although causal relationships remain incompletely defined. The immune system, therefore, should not be considered merely a bystander in cardiovascular disease. Antibody-mediated injury can occur through multiple, well-established mechanisms, including immune complex formation, complement activation, and recruitment of immune effector cells. Moreover, antibodies may bind directly to target antigens and trigger intracellular signaling, a mechanism well documented in other areas of immunology. A well-established example of activating autoantibodies is represented by anti–TSH receptor antibodies in Graves’ disease, which directly stimulate a G protein–coupled receptor, providing a prototypical model of agonistic autoimmunity [3].

Although agonistic activity is not unique to autoantibodies directed against the angiotensin II type 1 receptor (AT1R-AAs) and the endothelin-1 type A receptor (ETAR-AAs), these antibodies may represent a potential mechanism relevant to specific cardiovascular contexts, particularly those characterized by microvascular dysfunction and vascular remodelling, given their targeting of vasoactive G-protein–coupled receptors that play central roles in vascular tone, endothelial function, and tissue remodelling. Indeed, several other cardiac autoantibodies—including those directed against the β1-adrenergic receptor, the M2 muscarinic receptor, and specific ion channels—have been shown to exert agonistic or activating effects, predominantly influencing chronotropic, inotropic, or electrophysiological pathways. These examples should be regarded as precedents supporting the broader concept of agonistic autoimmunity in cardiovascular disease, rather than as distinct or opposing mechanisms, given that agonistic signaling has been clearly demonstrated for anti–β1-adrenergic receptor autoantibodies through activation of downstream pathways such as PKA [4]. In contrast, AT1R-AAs and ETAR-AAs preferentially engage vasoactive signaling pathways, are more consistently associated with vascular and microvascular phenotypes—such as hypertension, pulmonary arterial hypertension, and microvascular dysfunction—and induce sustained, ligand-independent receptor activation with limited desensitization. These features may confer distinct pathophysiological consequences at the vascular level compared with other forms of agonistic cardiac autoimmunity [5,6].

Instead of neutralizing antigens or tagging tissue for clearance, AT1R-AAs and ETAR-AAs function as persistent stimulators of two major vasoactive and profibrotic signaling pathways: the renin–angiotensin–aldosterone system, through AT1R activation, and the endothelin-1 pathway, through ETAR signaling [4,6]. This unique biology provides a conceptual framework linking immunology and cardiology, particularly in vascular-mediated cardiovascular injury. These mechanisms are most clearly supported in conditions characterized by microvascular dysfunction and vascular remodelling, and should not be generalized to all cardiovascular diseases. Beyond mediating receptor-driven signaling via established mechanisms, the immune system, in the presence of AT1R-AAs and ETAR-AAs, functions as an agonistic driver promoting vascular dysfunction and maladaptive cardiac remodelling. Importantly, in available clinical studies autoantibodies measurements were performed within hours of the acute event, a timeframe incompatible with de novo IgG production, indicating that detected AT1R-AAs and ETAR-AAs most likely reflect a pre-existing immunological status rather than a direct consequence of myocardial injury.

In this perspective, we delineate the mechanistic basis of AT1R- and ETAR-targeting agonistic autoantibodies, critically examine the emerging clinical evidence linking them to cardiovascular pathology, and define key priorities for their translational validation and potential therapeutic targeting.

## 2 A distinct biology

AT1R and ETAR are G-protein–coupled receptors that play central roles in vascular tone, endothelial function, and tissue remodelling. Their physiological ligands—angiotensin II and endothelin-1—are normally subject to strict spatial and temporal regulation. In contrast, AT1R-AAs and ETAR-AAs bypass this homeostatic control, driving sustained receptor activation [6,7]. Most mechanistic insights derive from experimental and translational models, and their quantitative contribution in human cardiovascular disease remains to be fully established. Beyond direct receptor activation, accumulating evidence shows that AT1R-AAs and ETAR-AAs trigger a broad range of pathogenic mechanisms. Immune complex formation and engagement of Fcγ receptors on endothelial cells, neutrophils, and monocytes potentiate proinflammatory signaling and amplify vascular injury. Intracellularly, sustained stimulation activates MAPK/ERK and NF-κB pathways, driving oxidative stress, endothelial activation, and cytokine release. In addition, AT1R activation by agonistic autoantibodies may involve biased signalling mechanisms, including a shift from canonical Gq-mediated pathways toward β-arrestin–dependent signalling. This switch may contribute to sustained MAPK/ERK activation and has been implicated in vascular remodelling, inflammation, and myocardial responses [[8], [9], [10], [11]].

**Fig. 1:**
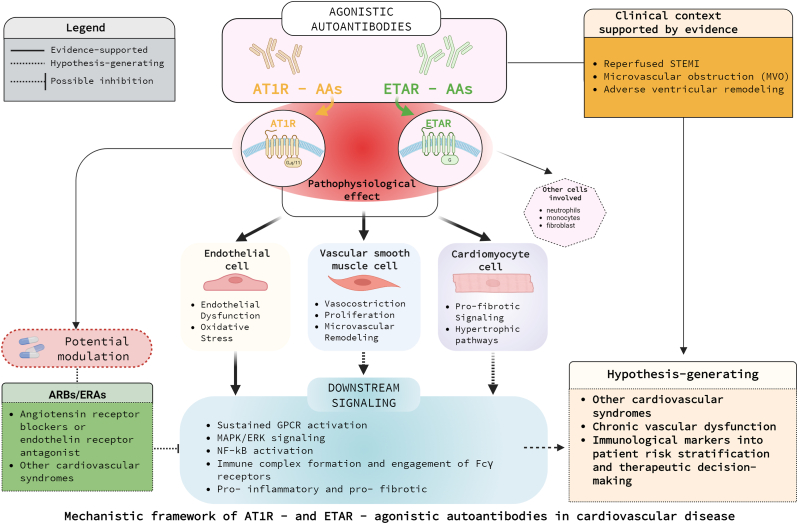
**Mechanistic framework of AT1R- and ETAR-agonistic autoantibodies in cardiovascular disease**. The schematic illustrates the proposed mechanisms by which agonistic autoantibodies targeting the angiotensin II type 1 receptor (AT1R-AAs) and the endothelin-1 type A receptor (ETAR-AAs) may contribute to cardiovascular pathology. Binding of autoantibodies to AT1R and ETAR on endothelial cells, vascular smooth muscle cells, and cardiomyocytes results in sustained receptor activation and downstream signaling, including vasoconstriction, endothelial dysfunction, oxidative stress, and pro-inflammatory and pro-fibrotic pathways. The clinical context in which these mechanisms are supported by direct evidence—reperfused ST-elevation myocardial infarction with microvascular obstruction—is shown separately from hypothesis-generating extensions to other cardiovascular settings. Potential points of pharmacological modulation (e.g., angiotensin receptor blockers or endothelin receptor antagonists) are indicated and explicitly labeled as hypothetical where direct clinical evidence is lacking. Solid lines indicate evidence-supported mechanisms, whereas dashed lines denote hypothesis-generating concepts.

In cardiac fibroblasts, these cascades promote matrix deposition and fibrosis, whereas in vascular smooth muscle cells they induce proliferation and neointimal hyperplasia. Together, these findings support the concept that functional autoantibodies exert complex biological actions extending beyond receptor agonism, coupling Fc receptor–mediated inflammation with maladaptive cardiovascular remodelling [4,5]. (Fig. 1) What emerges is not merely an additional autoantibodies-mediated mechanism, but a conceptual shift: immune responses acting as chronic receptor agonists, bridging classical neurohormonal activation with immune-driven vascular injury. This functional autoimmunity framework distinguishes AT1R-AAs and ETAR-AAs from previously described cardiomyocyte-directed autoantibodies in terms of receptor specificity and predominant vascular pathophysiological context, rather than agonistic capacity per se.

Much of the most robust mechanistic and translational evidence regarding agonistic autoantibodies targeting GPCRs derives from clinical settings such as preeclampsia, hypertension, and kidney transplantation, where AT1R- and ETAR-directed autoantibodies have been extensively characterized and, in some cases, integrated into clinical paradigms. Among these, preeclampsia represents one of the most extensively studied and clinically relevant conditions in which AT1R-targeting agonistic autoantibodies have been implicated. In this setting, AT1R-AAs have been shown to induce receptor activation, promote endothelial dysfunction, and contribute to vascular and inflammatory alterations, providing strong evidence of their pathogenic potential.

Moreover, preeclampsia has served as a key translational model in which critical aspects of receptor targeting have been elucidated, including epitope specificity, sustained agonistic activity, and downstream signalling pathways. More broadly, these extracardiac contexts have provided important insights into how functional autoantibodies can induce vascular dysfunction and inflammatory responses through persistent receptor activation [[12], [13], [14], [15]].

Accordingly, these conditions may be regarded as paradigmatic models that establish biological plausibility and offer a mechanistic foundation for exploring similar processes in cardiovascular disease. However, these findings should not be interpreted as direct evidence in cardiovascular settings, but rather as mechanistic precedents requiring phenotype-specific validation.

## 3 Reframing clinical puzzles

Recognition of these agonistic autoantibodies can provide an additional explanatory framework for unresolved issues in cardiology. However, available evidence is derived from observational associations and does not establish causality. In patients with ST-elevation myocardial infarction, restoration of epicardial patency does not always ensure adequate myocardial reperfusion. Coronary microvascular obstruction, often referred to as the ‘no-reflow’ phenomenon, remains a frequent complication, affecting up to 40% of patients and contributing to larger infarcts and worse outcomes. Although mechanisms such as microembolization, oedema, and haemorrhage have been implicated, observational studies have reported that AT1R-AAs and ETAR-AAs may be associated with impaired reperfusion and microvascular injury [16]. Because IgG autoantibodies were assessed within 12 h after PCI, their presence cannot be interpreted as a consequence of myocardial ischemia or reperfusion injury. Rather, these antibodies were almost certainly pre-existing and may identify patients with heightened susceptibility to microvascular injury and adverse remodelling. Their presence has further been linked to adverse ventricular remodelling and an increased risk of major adverse cardiovascular events, thereby supporting the hypothesis that persistent receptor overstimulation contributes to heterogeneous recovery following myocardial infarction [16].

This perspective may help generate hypotheses to explain why some patients progress to heart failure despite receiving guideline-directed medical therapy, including angiotensin receptor blockers, reflecting interindividual variability in treatment response rather than absence of standard-of-care therapies.

## 4 Beyond acute ischemia

Beyond the acute ischemic setting, interest in AT1R-AAs and ETAR-AAs may be extended toward cardiovascular conditions in which microvascular dysfunction, endothelial activation, and abnormal vasoactive signaling play a central pathophysiological role. Notably, evidence from conditions such as preeclampsia [17] and systemic sclerosis [12] further supports the pathophysiological role of autoantibodies targeting GPCRs, providing additional mechanistic insight from well-characterized immune-mediated diseases. On this basis, syndromes such as myocardial infarction with nonobstructive coronary arteries (MINOCA) and heart failure with preserved ejection fraction (HFpEF), which are characterized by coronary microvascular and endothelial dysfunction, could represent clinical settings in which functional autoimmunity targeting vasoactive receptors might be involved. However, it is important to emphasize that, to date, no clinical studies have directly investigated the presence or pathogenic role of AT1R-AAs or ETAR-AAs in patients with MINOCA or HFpEF. Accordingly, any implication of a demonstrated association in these conditions is currently unsupported by direct evidence.

Beyond vasoactive GPCR-targeting autoantibodies, chemokine receptor–directed autoimmunity may represent an additional mechanism linking immune activation with vascular dysfunction. In particular, the CXC-motif chemokine receptor 3 (CXCR3) plays a central role in leukocyte recruitment and inflammatory signaling, processes that are critically involved in endothelial injury and microvascular remodelling. In this context, population-based evidence from the Gutenberg Health Study [18], including approximately 5000 participants, supports the potential clinical relevance of autoantibodies targeting chemokine receptors. While these findings further support the broader concept of functional autoimmunity in cardiovascular disease, CXCR3-targeting autoantibodies should currently be regarded as complementary and hypothesis-generating, as direct evidence in cardiovascular clinical settings remains limited. Accordingly, the present perspective maintains its primary focus on agonistic autoantibodies targeting AT1R and ETAR.

The only clinical context in which AT1R-AAs and ETAR-AAs have been linked to myocardial injury remains reperfused ST-elevation myocardial infarction (STEMI), specifically in relation to microvascular obstruction (MVO) following percutaneous coronary intervention [16]. In this setting, MVO represents an acute ischemia–reperfusion injury occurring in the presence of obstructive coronary artery disease and is driven by mechanisms such as endothelial swelling, distal embolization, inflammatory activation, and microvascular collapse. Importantly, MINOCA and HFpEF are heterogeneous clinical syndromes characterized by fundamentally different substrates, temporal evolution, and diagnostic definitions. MINOCA, by definition, occurs in the absence of obstructive coronary artery disease and encompasses multiple underlying mechanisms, including plaque disruption, coronary spasm, embolization, and nonischaemic myocardial injury. HFpEF represents a chronic, systemic syndrome involving complex interactions among vascular stiffness, inflammation, metabolic dysregulation, and myocardial remodelling. Microvascular abnormalities described in these conditions therefore cannot be considered mechanistically equivalent to post-reperfusion MVO observed in STEMI patients. For these reasons, direct translation of antibody-associated MVO findings in reperfused STEMI to MINOCA or HFpEF is not currently justified. Where broader implications are discussed, they should be regarded as hypothesis-generating rather than evidence-based. Dedicated, phenotype-specific studies will be required to determine whether agonistic autoantibodies targeting AT1R or ETAR play any role outside the context of reperfused myocardial infarction.

## 5 Agonistic autoantibodies and myocardial pathophysiology

While AT1R-AAs and ETAR-AAs have been primarily investigated in vascular and microvascular contexts, increasing evidence suggests that their effects may extend to myocardial structure and function through both direct and indirect mechanisms. Chronic activation of vasoactive GPCRs signaling may promote myocardial remodelling by increasing afterload, impairing coronary microvascular perfusion, and sustaining low-grade inflammatory signaling within the cardiac interstitium [19].

At the myocardial level, sustained GPCRs activation driven by agonistic autoantibodies may activate pro-fibrotic and hypertrophic signaling pathways, including MAPK/ERK and TGF-β–related cascades, in both cardiomyocytes and cardiac fibroblasts. In parallel, endothelial dysfunction induced by AT1R- and ETAR-autoantibodies may impair endothelial–cardiomyocyte cross-talk, thereby exacerbating ischemia, oxidative stress, and maladaptive remodelling even in the absence of obstructive coronary disease [20].

Although direct clinical evidence linking AT1R-AAs and ETAR-AAs to specific myocardial syndromes remains limited, independent experimental and translational studies provide converging support for a potential myocardial impact of sustained vasoactive GPCRs activation. Models of chronic AT1R and ETAR signaling have consistently demonstrated pro-fibrotic remodelling, microvascular rarefaction, and impaired myocardial perfusion—mechanisms highly relevant to the development of heart failure and ischemia-related phenotypes. While these data do not establish disease-specific associations, they strengthen the biological plausibility of myocardial involvement beyond vascular compartments [21].

Nevertheless, several key questions remain unresolved. The relative contribution of direct myocardial effects versus indirect vascular-mediated mechanisms has not been systematically addressed in humans, and interventional data in antibody-positive patients are lacking. Whether modulation of agonistic autoantibodies activity can alter myocardial remodelling trajectories therefore remains a critical unanswered question.

## 6 Clinical implications

The identification of AT1R-AAs and ETAR-AAs carries potential clinical implications that extend beyond mechanistic insights. Although angiotensin receptor blockers are a cornerstone of guideline-directed medical therapy, their ability to counteract agonistic autoantibody–driven AT1R activation may vary among patients, potentially depending on antibody titer, epitope specificity, and functional activity. Importantly, pharmacological blockade of ligand-dependent AT1R activation may not fully suppress sustained or biased signaling induced by agonistic autoantibodies, particularly at standard doses used in clinical practice. Accordingly, any potential mitigating effect of ARBs on autoantibody-driven receptor activation should be interpreted with caution and remains to be tested in prospective, antibody-stratified studies. At present, such considerations are primarily supported by data from reperfused STEMI cohorts and should not be extrapolated to other cardiovascular syndromes in the absence of direct evidence [16]. Notably, the receptors targeted by these autoantibodies represent established pharmacological targets. Preclinical and clinical data suggest that angiotensin receptor blockers and endothelin receptor antagonists can mitigate antibody-driven receptor activation, raising the possibility that existing therapies could be repurposed for antibody-positive patients [6]. While this hypothesis remains to be confirmed in prospective, controlled studies, it provides a compelling rationale to incorporate immunological markers into patient risk stratification and therapeutic decision-making.

Beyond receptor blockade, the concept of functional autoantibodies invites broader exploration of immunomodulation. Could B-cell–directed therapies, Fc receptor blockade, or targeted immunoadsorption play a role in selected cardiovascular settings? Lessons from rheumatology and transplantation suggest they might. Importantly, these potential implications should be interpreted in light of the current limitations of the evidence base, which remains predominantly observational.

## 7 Challenges ahead

Several key limitations must be resolved before functional autoantibodies can be integrated into routine clinical practice. A major challenge lies in laboratory methodology: current assays lack harmonization, positivity thresholds differ across platforms, and results often fail to reflect biological activity, as antibody titers do not consistently correlate with functional agonistic effects. This highlights the urgent need for harmonized, functionally validated platforms that can quantify both antibody presence and biological activity. Currently available assays for AT1R- and ETAR-targeting autoantibodies vary substantially in methodology, functional readout, and clinical applicability (Table 1). Developing harmonized and reproducible, clinically validated assay frameworks will be essential to ensure comparability across studies and to enable reliable risk stratification in clinical settings. As highlighted, most available assays remain research-oriented, with limited harmonization and lack of functional validation, thereby restricting their integration into routine clinical practice. Regulatory pathways, including IVDR (In Vitro Diagnostic Regulation) compliance, will require robust analytical and clinical validation before widespread adoption.

**Table 1:** Current assay approaches for detection of AT1R- and ETAR-targeting autoantibodies.

Assay type	Platform	Availability	Functional assessment	Clinical applicability	Regulatory status
ELISA-based assays	Solid-phase immunoassay (commercial or in-house)	Commercial kits and research labs	No (binding only)	Limited; not routinely used in cardiology	Not IVDR-approved
Cell-based functional assays	GPCR-expressing cell lines (e.g., reporter assays)	Specialized research laboratories	Yes (agonistic activity)	Experimental; not standardized for clinical use	Not IVDR-compliant
Bioassays (e.g., cardiomyocyte or vascular cell response)	In vitro functional systems	Limited to expert centers	Yes (physiological response)	Research only	Not applicable
Multiplex immunoassays	Bead-based or multi-analyte platforms	Emerging, mostly research	No or limited	Not validated for routine use	Not IVDR-approved

AT1R, angiotensin II type 1 receptor; ETAR, endothelin type A receptor; GPCR, G protein–coupled receptor; ELISA, enzyme-linked immunosorbent assay; IVDR, In Vitro Diagnostic Regulation.

An additional limitation of the available clinical studies is that patients with underlying autoimmune diseases were not systematically excluded or stratified. Given that AT1R-AAs and ETAR-AAs have been described in several autoimmune conditions, this raises the possibility of confounding due to pre-existing immune dysregulation, which cannot be fully accounted for in current observational cohorts.

At present, the available evidence is largely derived from experimental models and observational studies and does not establish a causal relationship between functional autoantibodies and cardiovascular outcomes. Therefore, the proposed mechanisms should be interpreted as hypothesis-generating and require validation in prospective, phenotype-specific studies [16]. Large, multicenter investigations are urgently needed to define the prevalence of these antibodies across populations and to control for key confounders, including age, sex, and comorbidities.

The critical next step will be prospective interventional trials targeting antibody-positive patients. These studies are necessary to determine whether therapeutic approaches—from receptor blockade to immunomodulation—can mitigate the harmful effects of these autoantibodies and translate into improved clinical outcomes. These limitations underscore the need for caution in terms of accuracy, interpretation, and claims of novelty, while highlighting the importance of maintaining internal consistency when integrating immunological concepts into cardiovascular paradigms.

## 8 Translational outlook

At present, the available evidence is largely derived from specific clinical contexts, particularly reperfused ST-elevation myocardial infarction, and remains limited in other cardiovascular settings. Therefore, the broader applicability of this mechanism across the spectrum of cardiovascular disease should be considered hypothesis-generating and requires phenotype-specific validation.

Growing recognition of the pathogenic role of functional autoantibodies has opened new translational perspectives in cardiovascular medicine. Rather than novel experimental targets, the implicated receptors are already well established in clinical pharmacology, offering a unique opportunity to evaluate whether receptor blockade or broader immunomodulatory approaches can mitigate their deleterious effects. Future efforts should focus on translating these mechanistic insights—currently supported primarily in the context of reperfused myocardial infarction—into clinical benefit through carefully designed, phenotype-specific studies, prioritizing pre-event sampling, longitudinal antibody profiling, and careful exclusion or stratification of autoimmune conditions to disentangle baseline immunological risk from disease-related effects.

## 9 Conclusions

AT1R-AAs and ETAR-AAs expand the traditional view of autoantibodies in cardiovascular disease as passive markers or secondary by-products of tissue injury. By functioning as persistent, ligand-independent stimulators of vasoactive G-protein–coupled receptors, AT1R-AAs and ETAR-AAs support a mechanistic link between immune dysregulation and chronic neurohormonal activation, with particular relevance for vascular and microvascular pathology. Current evidence—largely derived from experimental models and observational clinical studies in reperfused ST-elevation myocardial infarction—supports an association between these autoantibodies and adverse microvascular injury and ventricular remodelling. Importantly, available data suggest that detected autoantibodies most likely reflect a pre-existing immunological substrate, reinforcing their potential role as markers of susceptibility rather than direct consequences of myocardial injury. At the same time, the lack of direct evidence in other cardiovascular syndromes underscores the need for caution when extrapolating these findings beyond data-supported contexts.

Rather than redefining cardiovascular disease through an immunological lens alone, the concept of functional autoimmunity proposed here offers a complementary framework that integrates immune-mediated receptor activation into established vascular and cardiac pathophysiology.

This perspective integrates mechanistic insights from experimental and extracardiac clinical settings to propose a biologically grounded, yet hypothesis-generating, framework for functional autoimmunity in cardiovascular disease. While this framework provides a biologically plausible link between immune dysregulation and cardiovascular pathology, its applicability across different cardiovascular phenotypes remains to be established and requires further validation.

In summary, AT1R-AAs and ETAR-AAs represent a relevant example of how immune mechanisms may intersect with classical cardiovascular pathways. Their study invites a more nuanced dialogue between immunology and cardiology—one grounded in mechanistic insight, tempered by methodological rigor, and oriented toward carefully validated translational progress. This perspective does not aim to establish causality but rather to integrate available experimental and clinical observations into a hypothesis-generating conceptual framework.

## CRediT authorship contribution statement

**Francesco Tona:** Writing – original draft, Supervision, Conceptualization. **Giacomo Bernava:** Writing – original draft. **Giovanni Civieri:** Writing – review & editing, Validation, Conceptualization.

## Declaration of competing interest

The authors declare that they have no competing financial interests or personal relationships that could have influenced the work reported in this manuscript. The authors received no specific funding for the preparation of this article.

## Data Availability

No data was used for the research described in the article.
